# Enhanced Cardiomyocyte Function in Hypertensive Rats With Diastolic Dysfunction and Human Heart Failure Patients After Acute Treatment With Soluble Guanylyl Cyclase (sGC) Activator

**DOI:** 10.3389/fphys.2020.00345

**Published:** 2020-05-25

**Authors:** Detmar Kolijn, Árpád Kovács, Melissa Herwig, Mária Lódi, Marcel Sieme, Abdulatif Alhaj, Peter Sandner, Zoltán Papp, Peter H. Reusch, Peter Haldenwang, Ines Falcão-Pires, Wolfgang A. Linke, Kornelia Jaquet, Sophie Van Linthout, Andreas Mügge, Carsten Tschöpe, Nazha Hamdani

**Affiliations:** ^1^Department of Molecular and Experimental Cardiology, Ruhr University Bochum, Bochum, Germany; ^2^Department of Clinical Pharmacology, Ruhr University Bochum, Bochum, Germany; ^3^Department of Cardiology, St. Joseph Hospital, Ruhr University Bochum, Bochum, Germany; ^4^Institute of Physiology, Ruhr University Bochum, Bochum, Germany; ^5^Division of Clinical Physiology, Department of Cardiology, Faculty of Medicine, University of Debrecen, Debrecen, Hungary; ^6^University of Debrecen, Kálmán Laki Doctoral School, Debrecen, Hungary; ^7^Bayer AG, Drug Discovery Cardiology, Wuppertal, Germany; ^8^Department of Cardiothoracic Surgery, University Hospital Bergmannsheil Bochum, Bochum, Germany; ^9^Department of Surgery and Physiology and Cardiovascular Research Centre, Faculty of Medicine, University of Porto, Porto, Portugal; ^10^Institute of Physiology II, University Hospital Münster, University of Münster, Münster, Germany; ^11^Department of Medicine and Cardiology (CVK), Charité-Universitätsmedizin Berlin, Berlin, Germany

**Keywords:** oxidative stress, inflammation, HFPEF, sGC activator, titin

## Abstract

**Aims:**

Our aim was to investigate the effect of nitric oxide (NO)-independent activation of soluble guanylyl cyclase (sGC) on cardiomyocyte function in a hypertensive animal model with diastolic dysfunction and in biopsies from human heart failure with preserved ejection fraction (HFpEF).

**Methods:**

Dahl salt-sensitive (DSS) rats and control rats were fed a high-salt diet for 10 weeks and then acutely treated *in vivo* with the sGC activator BAY 58-2667 (cinaciguat) for 30 min. Single skinned cardiomyocyte passive stiffness (F_passive_) was determined in rats and human myocardium biopsies before and after acute treatment. Titin phosphorylation, activation of the NO/sGC/cyclic guanosine monophosphate (cGMP)/protein kinase G (PKG) cascade, as well as hypertrophic pathways including NO/sGC/cGMP/PKG, PKA, calcium–calmodulin kinase II (CaMKII), extracellular signal-regulated kinase 2 (ERK2), and PKC were assessed. In addition, we explored the contribution of pro-inflammatory cytokines and oxidative stress levels to the modulation of cardiomyocyte function. Immunohistochemistry and electron microscopy were used to assess the translocation of sGC and connexin 43 proteins in the rat model before and after treatment.

**Results:**

High cardiomyocyte F_passive_ was found in rats and human myocardial biopsies compared to control groups, which was attributed to hypophosphorylation of total titin and to deranged site-specific phosphorylation of elastic titin regions. This was accompanied by lower levels of PKG and PKA activity, along with dysregulation of hypertrophic pathway markers such as CaMKII, PKC, and ERK2. Furthermore, DSS rats and human myocardium biopsies showed higher pro-inflammatory cytokines and oxidative stress compared to controls. DSS animals benefited from treatment with the sGC activator, as F_passive_, titin phosphorylation, PKG and the hypertrophic pathway kinases, pro-inflammatory cytokines, and oxidative stress markers all significantly improved to the level observed in controls. Immunohistochemistry and electron microscopy revealed a translocation of sGC protein toward the intercalated disc and t-tubuli following treatment in both control and DSS samples. This translocation was confirmed by staining for the gap junction protein connexin 43 at the intercalated disk. DSS rats showed a disrupted connexin 43 pattern, and sGC activator was able to partially reduce disruption and increase expression of connexin 43. In human HFpEF biopsies, the high F_passive_, reduced titin phosphorylation, dysregulation of the NO–sGC–cGMP–PKG pathway and PKA activity level, and activity of kinases involved in hypertrophic pathways CaMKII, PKC, and ERK2 were all significantly improved by sGC treatment and accompanied by a reduction in pro-inflammatory cytokines and oxidative stress markers.

**Conclusion:**

Our data show that sGC activator improves cardiomyocyte function, reduces inflammation and oxidative stress, improves sGC–PKG signaling, and normalizes hypertrophic kinases, indicating that it is a potential treatment option for HFpEF patients and perhaps also for cases with increased hypertrophic signaling.

## Introduction

Despite major therapeutic advances in the treatment of heart failure (HF) in general, the subtype HF with preserved ejection fraction (HFpEF) continues to take a significant clinical toll in terms of morbidity and mortality. Current treatment options for HFpEF patients are limited, and no drug has yet been shown to improve diastolic function in HFpEF patients. Many of the tested compounds were ineffective in reducing morbidity and mortality, and numerous clinical trials have failed to show that the drug under investigation delivered any positive effect. Patient management is presently limited to amelioration of symptoms and the treatment of common comorbidities such as hypertension, diabetes, obesity, and atrial fibrillation. The recent outcome of the PARAGON-HF trial showed evidence of a heterogeneous response to treatment, with potential benefit in certain subgroups such as women and patients with an EF below the median. These data strongly imply that “one size might not fit all” in HFpEF (ClinicalTrials.gov Identifier: NCT0192071). Many studies from our and other groups have suggested that boosting the cyclic guanosine monophosphate (cGMP)-dependent protein kinase or protein kinase G (cGMP–PKG) pathways is a promising target when aiming to improve diastolic function in HFpEF patients (Bishu et al., 2011; Hamdani et al., 2013a, 2014). Studies have shown that PKG is reduced in HFpEF patients and animal models probably due to inflammation and oxidative stress, thus suggesting that it is a possible treatment target in patients (Franssen et al., 2016). Unfortunately, the RELAX trial using a phosphodiesterase (PDE)5 inhibitor to increase cGMP failed to show any beneficial effect in HFpEF patients (van Heerebeek et al., 2012), as inhibition of PDE5 did not appear to improve the low cGMP content found in HFpEF (Redfield et al., 2013). In the >100 HFpEF patients studied, sildenafil failed to raise plasma cGMP or ameliorate diastolic left ventricular (LV) dysfunction. The authors suggested that these disappointing results were (partially) attributable to the relatively low right-sided heart pressures in their patient group compared with earlier studies in HF with reduced EF (HFrEF); moreover, PDE5 was undetectable in humans or experimental models of HF lysates, whereas it is present in the murine and bovine lung samples which were used as a positive control (Degen et al., 2015). These results indicate that if PDE5 is expressed in cardiac tissue, it is present in very low quantities. In addition, based on the elevated basal plasma levels of N-terminal pro-B type natriuretic peptide (NT-proBNP) and the high prevalence of atrial fibrillation, it seems that patients in the RELAX trial were at an advanced stage of HFpEF and therefore less likely to benefit from a limited strategy involving only inhibition of cGMP breakdown *via* PDE5. In particular, PDE9 was recently shown to be upregulated in hypertrophy and cardiac failure. PDE9 is expressed in the mammalian heart (including human) and regulates NP rather than NO-stimulated cGMP in cardiomyocytes (Lee et al., 2015), and its inhibition protects against pathological responses to neurohormones *in vitro* and sustained pressure overload stress *in vivo*. PDE9 expression is increased in the LV of patients with hypertrophy due to aortic stenosis (pressure overload), and even more so in HFpEF patients. These data suggest that inhibition of PDE9 activated PKG and might blunt pathological stress responses. Both PDE5 and PDE9 regulate cGMP–PKG activity, and in combination, they could be beneficial in the treatment of HFpEF. Given the possibility that changes in cGMP may be an important underlying pathophysiologic mechanism in HFpEF, stimulation of cGMP production remains an interesting therapeutic strategy in HFpEF. However, boosting myocardial cGMP levels might be possible by alternative means, and many different strategies for cGMP enhancement are available. The soluble guanylyl cyclase (sGC) stimulators and activators may have a particular therapeutic potential in cardiovascular disease (Evgenov et al., 2006), and the effect of both depends on the oxidation state and heme content of sGC, and both have been shown to be effective in increasing the pool of cGMP (Evgenov et al., 2006). sGC stimulators, such as BAY 41–2272, BAY 41-8543, BAY 63-2521 (riociguat), and BAY 1021189 (vericiguat), increase sGC activity of the native non-oxidized and heme-containing sGC. Thus, sGC stimulators are NO-independent but heme-dependent. In contrast, sGC activators such as Bay 58-2667 (cinaciguat) increase enzyme activity when the heme iron is oxidized (Fe3+) or the heme group is missing (Stasch et al., 2001, 2011; Sandner et al., 2019), and they are termed NO-independent and heme-independent sGC activators. Under physiological conditions, sGC exists in equilibrium between its reduced and oxidized state, but oxidative stress shifts this equilibrium toward the NO-insensitive ferric/heme-free form (Stasch et al., 2006). In line with these findings, cinaciguat relaxed blood vessels in diseased animal models including hypertensive rats and hyperlipidemic rabbits, more efficacious compared to control, healthy animals. These effects are enhanced in the presence of oxidized sGC (Stasch et al., 2006).

Studies of sGC stimulators and activators in experimental models of hypertension have provided valuable insights into their therapeutic potential. These drugs induced vasodilation, attenuated cardiac fibrosis and hypertrophy, normalized blood pressure, protected against cardiac and renal damage, and improved survival in rat models of hypertension (Mittendorf et al., 2009; Stasch and Hobbs, 2009; Wilck et al., 2018). Beneficial effects were also observed in patients with pulmonary arterial hypertension (Stasch et al., 2011). In addition, other potential effects in HF may involve the prevention of myocardial dysfunction, dysregulation of the NO–sGC–cGMP pathway, and increased diastolic stiffness associated with endothelial dysfunction. Augmenting sGC activity might therefore decrease titin-based stiffness through increased cardiomyocyte cGMP and thus counteract myocardial stiffening in HF, particularly in HFpEF.

A clinical trial using the sGC stimulator, vericiguat, showed that the compound was well tolerated when given once a day to patients with HFrEF, leading to lower levels of N-terminal pro-B type natriuretic peptide (NT-proBNP) (trial SOCRATES-REDUCED) (Gheorghiade et al., 2016). Vericiguat was also tested in HFpEF patients in the SOCRATES-PRESERVED trial, a phase IIb study of NO–sGC stimulation with vericiguat for 12 weeks. The outcome was improved quality of life without changing NT-proBNP levels, thus encouraging further studies to boost the NO-sGC pathway in HFpEF patients (Pieske et al., 2017).

The present study investigated the effects of an sGC activator in Dahl salt-sensitive (DSS) rats exhibiting hypertension and diastolic dysfunction and was designed to examine the underlying mechanisms and pathways that contribute to cGMP decline in HFpEF. Since both NO and sGC levels are reduced in HFpEF, we hypothesized that treatment with the sGC activator BAY 58-2667 would improve the functioning of this pathway and thereby improve cardiomyocyte function *via* enhanced titin phosphorylation.

## Methods

### Human Studies

All procedures were performed according to the Declaration of Helsinki and were approved by the local ethics committee. Biopsies were obtained for the primary purpose of diagnosis following ethics committee approval (EA2/140/16) and informed consent. Control samples were obtained from explanted donor hearts following ethics committee approval (OKAR/1066/2008/OKAR). Effects of *in vitro* incubation of sGC stimulation on cardiomyocyte passive mechanics and titin phosphorylation were studied on LV myocardial samples obtained from biopsies from HFpEF patients (*n* = 14/samples; for patient characteristics, see Table 1) as well as from healthy donors (*n* = 10/samples).

**TABLE 1:** HFpEF patient characteristics.

**HFpEF patients**	
***n***	**14**

Age, years (mean ± SD)	61.5 ± 14.5
EF, % (mean ± SD)	65.6 ± 11.6
NYHA class	II–III
LV EDP, mmHg (mean ± SD)	16.3 ± 6.3
NT-proBNP, ng/L (mean ± SD)	520 ± 440
E/E’	16.8 ± 4.6
ACE inhibitors (%)	76.0
Diuretics (%)	66.5
Digoxin (%)	12.5
AT_1_ receptor antagonists (%)	30.1
Aldosterone antagonists (%)	13.5
Amiodarone (%)	19.5
Beta-blockers (%)	80.0

*EDP, end-diastolic pressure; EF, ejection fraction; LV, left ventricular; NT-proBNP, N-terminal pro-B type natriuretic peptide; NYHA, New York Heart Association; E/E’, mitral inflow E wave/tissue Doppler mitral annulus velocity; HFpEF, heart failure with preserved ejection fraction.*

HFpEF patients were referred for cardiac catheterization and endomyocardial biopsy procurement because of clinical suspicion of a cardiomyopathy. They all had been admitted to hospital because of worsening HF. Subsequent histological examination ruled out a cardiomyopathy/infiltrative myocardial disease. Coronary angiography showed the absence of significant coronary artery stenosis or graft vasculopathy. All patients satisfied the criteria as proposed by the European study group on HFpEF, i.e., signs and symptoms of congestive HF, LV ejection fraction (EF) ≥ 50% and abnormal LV end-diastolic pressure at rest > 12 mmHg (van Heerebeek et al., 2006, 2008, 2012). All patients had one or more predisposing risk factors for diastolic LV dysfunction. Endomyocardial biopsies were obtained from the LV and were snap frozen in liquid nitrogen and stored at −80°C until processing.

### Animal Model

Care and use of laboratory animals, as well as experimental procedures, were reviewed and approved by Ruhr University Bochum (Germany; ethical statement number: 84-02.04.2015.A449). Our study complied with the *Declaration of Helsinki* and with the *Directive 2010/63/EU of the European Parliament and of the Council “on the protection of animals used for scientific purposes*.*”* Male DSS rat (SS/JrHsdMcwiCrl; *n* = 55) and SS-13^BN^ (SS-Chr 13^BN^/McwiCrl; *n* = 55) consomic control strains were obtained from Charles River Laboratories (Wilmington, MA, United States). Eight-week-old DSS rats were fed a high-salt diet for 10 weeks to induce hypertension. Then, the rats were treated with BAY 58-2667 (obtained from Bayer AG, Wuppertal, Germany). Administration of sGC activator BAY 58-2667 was initiated at 18th week of age intravenously for 30 min. The animals (eight animals from each group) were housed on a 12/12 h light/dark cycle with constant temperature (22–23°C), with access to food and tap water *ad libitum*. Animals were anesthetized with either isoflurane (induction with 3%, maintenance with 1.5%), and the hearts were taken out and frozen in nitrogen.

### Force Measurements on Isolated Cardiomyocytes

Force measurements were performed on single demembranated cardiomyocytes (*n* = 12–42/5–6 heart/group) as described before^17^. Briefly, LV samples were de-frozen in relaxing solution (containing in mM: 1.0 free Mg^2+^; 100 KCl; 2.0 EGTA; 4.0 Mg-ATP; 10 imidazole; pH 7.0), mechanically disrupted and incubated for 5 min in relaxing solution supplemented with 0.5% Triton X-100 (all from Sigma-Aldrich). The cell suspension was washed five times in relaxing solution. Single cardiomyocytes were selected under an inverted microscope (Zeiss Axiovert 135, 40× objective; Carl Zeiss AG Corp., Obochen, Germany) and attached with a silicone adhesive between a force transducer and a high-speed length controller (piezoelectric motor) as part of a “Permeabilized Myocyte Test System” (1600A; with force transducer 403A; Aurora Scientific, Aurora, ON, Canada).

Cardiomyocyte Ca^2+^-independent passive force (F_passive_) was measured in relaxing buffer at room temperature within a sarcomere length (SL) range between 1.8 and 2.4 μm. Force values were normalized to myocyte cross-sectional area calculated from the diameter of the cells, assuming a circular shape. Subsequently, cardiomyocytes were incubated for 40 min in relaxing solution supplemented in one set with PKA and the other set with PKG1α (batch 034K1336, 0.1 U/ml; Sigma-Aldrich), cGMP (10 μM; Sigma-Aldrich), and dithiothreitol (DTT; 6 mM; Sigma-Aldrich). Thereafter F_passive_ measurements were again performed in relaxing solution at SL 1.8–2.4 μm.

Human LV samples were thawed in relaxing solution and incubated *in vitro* with 0.3 μM/L sGC stimulator for 30 min. Demembranation was performed following incubation with sGC stimulator. Cardiomyocyte (*n* = 12/group) F_passive_ was thereafter measured within an SL range between 1.8 and 2.4 μm as described above.

### Titin and Phospho-Titin Analysis by Western Blot

Polyacrylamide gel electrophoresis (PAGE) was performed to separate titin as previously described (Hamdani et al., 2013b). Briefly, LV tissue samples (*n* = 8–10/group) were solubilized in 50 mM Tris-sodium dodecyl sulfate (SDS) buffer (pH 6.8) containing 8 μg/ml leupeptin (Peptide Institute Inc., Ibaraki, Osaka, Japan) and 10 μl/ml phosphatase inhibitor cocktail (P2850; Sigma-Aldrich). Samples were heated for 3 min at 96°C and centrifuged. Samples were applied in duplicates at concentrations that were within the linear range of the detection system (20 and 25 μg dry weight; checked by spectroscopic methods) and separated by agarose-strengthened 1.8% SDS-PAGE. Gels were run at 4 mA constant current for 16 h. Thereafter, Western blot (WB) was performed to measure site-specific and total phosphorylation of titin. Following SDS-PAGE, proteins were transferred to polyvinylidene difluoride (PVDF) membranes (Immobilon-P 0.45 μm; Merck Millipore, Burlington, MA, United States). Blots were pre-incubated with 3% bovine serum albumin in Tween Tris-buffered saline (TTBS; containing 10 mM Tris-HCl; pH 7.6; 75 mM NaCl; 0.1% Tween; all from Sigma-Aldrich) for 1 h at room temperature. Then, blots were incubated overnight at 4°C with the primary antibodies.

Anti-phospho serine (Ser)/threonine (Thr) antibody (dilution 1:500; ECM Biosciences LLC, Versailles, KY, United States) was used to assess total titin phosphorylation. Phosphosite-specific anti-titin antibodies were custom-made by Eurogentec (Seraing, Belgium) with positions in N2Bus (N2B unique sequence) and PEVK (rich in proline, glutamate, valine, and lysine amino acids) domains of mouse (*Mus musculus*) titin according to UniProtKB identifier A2ASS6 (Hamdani et al., 2013b). The following rabbit polyclonal affinity purified antibodies were used:

– anti-phospho-N2Bus (**SER3991**) against EEGKS(PO3H2)LSFPLA (dilution 1:500);

– anti-phospho-N2Bus (**SER4043**) against QELLS(PO3H2)KETLFP (dilution 1:100);

– anti-phospho-N2Bus (**SER4080**) against LFS(PO3H2)EWLRNI (dilution 1:500);

– anti-phospho-PEVK (**SER12742**) against EVVLKS(PO3H2)VLRK (dilution 1:100);

– anti-phospho-PEVK (**SER12884**) against KLRPGS(PO3H2)GGEKPP (dilution 1:500).

The amino acid sequences of rat titin at Ser3991, Ser4043, Ser4080, Ser12742, and Ser12884 are identical to the amino acid sequences of mouse and refer to human titin at Ser4010, Ser4062, Ser4099, Ser11878, and Ser12022, respectively.

After washing with TTBS, primary antibody binding was visualized using secondary horseradish peroxidase (HRP)-labeled, goat anti-rabbit antibody (dilution 1:10,000; DakoCytomation, Glostrup, Denmark) and enhanced chemiluminescence (ECL Western Blotting Detection; Amersham Biosciences). WB signals were visualized using the LAS-4000 Image Reader and analyzed with Multi Gauge V3.2 software (both from FUJIFILM Corp., Minato, Tokyo, Japan). Coomassie-based PVDF stains were saved for comparison of protein load, and titin phosphorylation levels were indexed to the signal intensities obtained from PVDF staining. Finally, signals obtained from phospho-specific antibodies were normalized to signals obtained from PVDF stains referring to the entire protein amount transferred.

Human LV samples from HFpEF patients and donor controls (*n* = 8–10/group) were incubated *in vitro* with 1.0 μM/L sGC activator for 30 min. Subsequently, total titin phosphorylation of the sum of N2BA and N2B titin as Ser/Thr phosphorylation and PKA and PKG site-specific titin phosphorylation as Ser4010 and Ser4099 phosphorylation were assessed as described above.

For human phosphosite-specific anti-titin antibodies were custom-made by Eurogentec (Seraing, Belgium) with positions in N2Bus (N2B unique sequence) domains of human titin according to UniProtKB identifier Q8WZ42 (Hamdani et al., 2013b). The following rabbit polyclonal affinity purified antibodies were used:

– anti-phospho-N2Bus (**Ser4010**) against EEGKS(PO3H2)LSFPLA (dilution 1:500);

– anti-phospho-N2Bus (**Ser4099**) against QELLS(PO3H2)KETLFP (dilution 1:100).

### Myocardial Cyclic Guanosine Monophosphate Level

According to previous protocol (Franssen et al., 2016), myocardial cGMP was determined in LV homogenates (*n* = 8–10/group) by use of parameter cGMP assay immunoassay kit (R&D Systems, Minneapolis, MN, United States), in which cGMP present in the homogenate competes with a fixed amount of HRP-labeled cGMP for sites on a rabbit polyclonal antibody. The homogenates were diluted in cell lysis buffer, and 100 μl of 0.025-μg/μl protein aliquots were assayed according to manufacturer’s instructions. Results of duplicate determinations were averaged and expressed as μg/μl.

### Myocardial Protein Kinase G Activity

LV tissues samples (*n* = 8–10 samples) were homogenized in 25 mM Tris-HCl (pH 7.4), 1 mM EDTA, 2 mM EGTA, 5 mM DTT, 0.05% Triton X-100, and protease inhibitor cocktail (all from Sigma-Aldrich) and centrifuged for 5 min. Supernatants containing equal amounts of total protein were analyzed for PKG activity as described previously (Franssen et al., 2016). Briefly, reaction mixtures were incubated at 30°C for 10 min. Reaction mixtures contained 40 mM Tris-HCl (pH 7.4), 20 mM Mg(CH_3_COO)_2_, 0.2 mM [^32^P] adenosine triphosphate (ATP) (500–1,000 cpm pM^–1^; Amersham PLC, Little Chalfont, United Kingdom), 113 mg/ml heptapeptide (RKRSRAE), and 3 μM cGMP (both from Promega Corp., Madison, WI, United States), and a highly specific inhibitor of cyclic adenosine monophosphate-dependent protein kinase (5–24; Calbiochem, San Diego, CA, United States). The reaction was terminated by spotting 70 μl onto Whatman P-81 filters (MACHEREY-NAGEL). Samples were subsequently incubated and washed with 75 mM H_3_PO_4_ for 5 min to remove unbound ATP. Filters were then washed with 100% ethanol and air-dried before quantification. PKG activity was quantified using a Wallac 1409 Liquid Scintillation Counter (Hidex Oy, Turku, Finland). Specific activity of PKG was expressed as pM of ^32^P incorporated into the substrate (pM/min/mg protein).

### Myocardial Protein Kinase A and C Activity

PKA and PKC activity (*n* = 8–10 samples) was analyzed using non-radioactive PKA and PKC kinase activity assay kit (Enzo Life Science). Samples were homogenized in cell lysis buffer (20 mmol/L MOPS, 50 mmol/L β-glycerolphosphate, 50 mmol/L sodium fluoride, 1 mmol/L sodium vanadate, 5 mmol/L EGTA, 2 mmol/L EDTA, 1% NP40, 1 mmol/L DTT, 1 mmol/L benzamidine, 1 mmol/L phenylmethanesulphonylfluoride, and 10 μg/ml leupeptin and aprotinin, each). Supernatants were collected after centrifugation at 13,000 rpm for 30 min. Supernatants containing equal amounts of total protein (30 ng/μl protein aliquots were assayed according to manufacturer’s instructions) were added into the appropriate wells of the PKA and PKC substrates microliter plate. PKA and PKC kinase reaction was initiated by addition of ATP, and samples were subsequently incubated at 30°C for 90 min. Phosphorylated peptide substrates were recognized by phospho-specific substrate antibody. The phospho-specific antibody was subsequently bound by a peroxidase-conjugated secondary antibody anti-rabbit IgG:HRP. The assay was developed with tetramethylbenzidine, and the intensity of the color was measured in a microplate reader at 450 nm. Results of triplicate determinations were averaged, and specific activity of PKA was expressed as ng/μl.

### Myocardial Calcium–Calmodulin Kinase II Activity Testing

CaMKII activity (*n* = 8–10 samples) was determined using a CycLex^®^ CaMKII assay kit (CY-1173; MBL Corporation, MA, United States) according to the manufacturer’s guidelines. Briefly, frozen heart tissues were homogenized in sample buffer containing 15% glycerol, 62.5 mmol/L Tris; pH 6.8; 1% (w/v) SDS, protease inhibitor, and protein phosphatase inhibitor, all prepared in distilled H_2_O. Homogenates were centrifuged at 10,000 × g for 15 min at 4°C. The supernatant was removed and stored at -80°C. Protein samples were loaded onto microtiter wells (concentration, ∼2.0 μg/well) coated with CaMKII specific substrate, syntide-2, along with kinase reaction buffer with or without Ca^2+^/calmodulin. To quantify CaMKII activity, a standard curve correlating the amount of active CaMKII and the level of phosphorylation of syntide-2 was constructed.

### Myocardial Protein Kinase Extracellular Signal-Regulated Kinase 2

ERK2 activity (*n* = 8–10 samples) was analyzed using non-radioactive ERK2 kinase activity assay kit (CHEMICON). Homogenates were made in cell lysis buffer (50 mM Tris, pH 8.0, 150 mM NaCl, 0.5 mM EDTA, 1 mM DTT, 1% NP-40, 0.5% sodium deoxycholate, 0.1% SDS, 32 μM phenazine methosulphate (PMS), 200 nM aprotinin, 4 μM leupeptin, 100 μM sodium vanadate). Supernatants were collected after centrifugation at 12,000 rpm for 10 min at 4°C. Then, 20 μl of anti-MAP kinase antibody were added to 1 ml of cell lysate and incubated for 1–12 h at 4°C on a shaking or rocking platform. ERK2 kinase reactions were initiated by addition of 10 μl of 5× ATP/MgCl_2_ solution, and samples were subsequently incubated at 30°C for 60 min. Phosphorylated peptide substrates were recognized by phosphospecific substrate antibody. The enzyme reaction was terminated by adding 100 μl of stop solution to each well, including the control wells. Color intensity was measured in a microplate reader at 450 nm. Results of triplicate determinations were averaged.

### Immunofluorescence Imaging

Frozen LV unfixed slides (*n* = 3 samples/heart) were air-dried for 10 min and fixed in 4% paraformaldehyde in phosphate buffered saline (PBS; Sigma-Aldrich). After washing 3 times in PBS for 5 min, tissue was blocked in 5% bovine serum albumin (Sigma-Aldrich) in PBS for 1 h at room temperature. After further washing 3 times in PBS for 5 min, fixed slides were dual-stained with anti-guanylyl cyclase β1 ER-19 (Sigma-Aldrich; dilution 1:200) or Connexin 43 (Sigma-Aldrich; dilution 1:400/Thermofisher Scientific; dilution 1:200) antibodies and anti-α-actinin (sarcomere; Sigma-Aldrich; dilution 1:400) overnight at 4°C. After washing in PBS, slides were subsequently incubated overnight with secondary antibodies: fluorescein (FITC) anti-mouse (Rockland Immunochemicals Inc., Limerick, PA, USA; dilution 1:300) and Cy3 anti-rabbit (Jackson ImmunoResearch Laboratories Inc., West Grove, PA, USA; dilution 1:100). After multiple washings slides were covered and sealed by Mowiol mounting medium and ultrathin glass coverslips (Thermo Fisher Scientific). Immuno-stained samples were analyzed by confocal laser scanning microscopy (cLSM) (Nikon Eclipse Ti-E Inverted Microscope System; Nikon Instruments, Nikon Corp, Shinagawa, Tokyo, Japan). Immunofluorescence imaging parameters were identical among groups.

### Electron Microscopy

A small piece from the flash-frozen rat LV was cut and fixed in a 0.1 M PBS buffered fixative containing 4% paraformaldehyde (Sigma Aldrich, St. Louis, MO, United States) and 15% picric acid (Sigma Aldrich, St. Louis, MO, United States) overnight 4°C on a shaker. After washing the tissues three times in 0.1 M PBS and blocking with 20% normal goat serum (NGS) in PBS for 1 h, the primary antibody against PKG (Enzo Life Sciences, Farmingdale, United States) (1:200) and sGC (Sigma Aldrich, St. Louis, MO, United States) was used in a blocking solution (2% NGS in 0.1 M PBS) overnight 4°C on a shaker. After three times washing in 0.1 M PBS, the tissues were exposed to 1.4 nm nanogold conjugated anti-rabbit secondary antibody (Nanoprobes, NY, United States) (1:100) in the blocking solution overnight 4°C on shaker. After washing, the tissues were fixed in 1% glutaraldehyde in PBS for 10 min at room temperature. Silver enhancement was performed with HQ silver kit (Nanoprobes, NY, United States) after washing the samples with 0.1 M PBS and distillated water. After the enhancement, tissues were washed again, then treated with 0.5% osmium-tetroxide for 45 min, dehydrated in a grading series of ethanol and in propylene oxide, and embedded in DURCUPAN^TM^ ACM resin (Sigma Aldrich, St. Louis, MO, United States). Sections of 50 nm were cut, counterstained with UranyLess (Electron Microscopy Sciences, Hatfield, PA, United States) for 10 min, and investigated using a Zeiss LEO 910 electron microscope. Pictures were made using magnifications of 4,000× and 8,000×.

### Quantification of Tissue Oxidative Stress and Inflammation

Myocardial levels (*n* = 8–10 samples) of oxidative stress and inflammatory markers were tested using enzyme-linked immunosorbent assay (ELISA) and colorimetric assay kits: 3-nitrotyrosine ELISA kit (ab116691; Abcam), lipid peroxidation (malondialdehyde) assay kit (ab118970; Abcam), interleukin-6 (IL6) ELISA kit (ab100772; Abcam), intercellular cell adhesion molecule-1 (ICAM1) ELISA kit (ERICAM1; Thermo Fisher Scientific), vascular cell adhesion molecule-1 (VCAM1) ELISA kit (KHT0601; Thermo Fisher Scientific), and tumor necrosis factor alpha (TNFα) ELISA kit (ab108913; Abcam). Hydrogen peroxide (H_2_O_2_) was assessed in LV tissue homogenates (*n* = 8–10/group). Samples containing equal amounts of total protein were analyzed for H_2_O_2_ formation. H_2_O_2_ formation was measured by colorimetry at 540 nm. Results were converted using the standard curve for a known concentration of H_2_O_2_.

### Quantification of Tissue Nitric Oxide

The concentration of NO (*n* = 8–10 samples) was assessed by means of a colorimetric assay kit (BioVision Inc., Milpitas, CA, United States). This assay quantitates NO production by providing a measure of total nitrate/nitrite. NO production was measured in tissue homogenates. Briefly, LV tissue samples (*n* = 8–10/group) were treated with trichloroacetic acid (8 g in 80 ml acetone; Sigma-Aldrich) and washed with 1 ml 0.2% DTT. Tissue samples were homogenized in 1% SDS sample buffer [Tri-distilled water: 8.47 ml; glycerol: 2.1 ml; 10% SDS: 1.4 ml; 0.5 M Tris-HCl (pH 6.8): 1.75 ml; brome-phenol blue: 0.28 ml; DTT: 32.4 mg; all from Sigma-Aldrich]. These tissue samples underwent sonication and were subsequently centrifuged at 14,000 *g* for 15 min at 5°C. Supernatants containing equal amounts of total protein were analyzed for NO concentration.

In the first step, nitrate was converted to nitrite using nitrate reductase. In the second step, Griess reagents convert nitrite to an azochromophore reflecting NO concentration in the tissue samples. Nitrite levels could be measured independently from nitrate by omitting the first step. The absorbance of samples was measured at 540 nm using a plate reader. An assay buffer was used to generate a standard curve from which the absorbances of the samples could be translated into the nitrite and nitrate concentrations.

### Statistical Analysis

Descriptive statistics for data location and variability was based on arithmetic mean and standard error of the mean (SEM), respectively. Groups defined by disease status and levels of SL, or a combination of these factors, were compared in terms of continuous variables using Student’s two-sample t test (if distributional assumptions were satisfied) or Wilcoxon’s rank-sum test (otherwise). All such tests were unpaired (the effect of all factors including stimulation length was assessed on independent samples) and stratified for equivalence on other factors, if any. Differences were considered to be statistically significant at *P* < 0.05.

## Results

### Improved Diastolic Function Upon Soluble Guanylyl Cyclase Activator Treatment in Dahl Salt-Sensitive Rats

Transthoracic echocardiography was performed, and EF and fractional shortening were measured (parasternal long-axis view in M-mode) in anesthetized DAHL rats (*n* = 4) and controls (*n* = 4). Peak early (E), late (A) diastolic filling velocities and isovolumetric relaxation time (IVRT) were measured by pulsed wave Doppler. Tissue Doppler mode was applied to measure peak early diastolic (e′) and late diastolic (a′) mitral annular velocities. Echocardiographic measurements were performed at baseline conditions and after the treatment. Overall and despite the low n numbers, DAHL rats displayed a diastolic dysfunction indicated by prolonged IVRT, reduced E/A ratio. sGC activator significantly shortened IVRT and increased E/A ratio, indicating an improved diastolic function (Table 2).

**TABLE 2:** *In vivo* data before and after treatment with sGC activator in control and DSS groups.

	**Control**	**Control + sGC**	**DAHL**	**DAHL + sGC**
IVRT (ms)	28.25 ± 0.1	28.32 ± 0.1	34.44 ± 0.7***	28.46 ± 0.4^†⁣†⁣†^
E/A	2.0 ± 0.1	2.0 ± 0.02	1.2 ± 0.02***	1.44 ± 0.03^†⁣†⁣†^
e′/a′	1.6 ± 0.1	1.8 ± 0.05	1.24 ± 0.04	1.34 ± 0.03
EF	55.11 ± 1.1	54.8 ± 0.1	56.14 ± 3.61	53.5 ± 1.2
dP/dtmax (mmHg/s)	8.610 ± 211.6	8.334 ± 55.5	9.772 ± 122.0*	8.538 ± 163.7^†^
dP/dtmin (mmHg/s)	−10.578 ± 138.6	−10.201 ± 185	−8.423.25 ± 180*	−10.203 ± 357^†^
Tau (ms)	9.94 ± 1.8	10.17 ± 0.1	11.55 ± 0.7	9.8 ± 0.21^(^*^*p*^* ^= 0.06)^
TD (ms)	28.90 ± 1.2	27.30 ± 1.0	37.70 ± 1.0**	26.95 ± 0.96^†⁣†^

LV, left ventricular; IVRT, isovolumetric relaxation time; A, peak trans-mitral late left LV diastolic filling velocity; E, peak trans-mitral early LV diastolic filling velocity; a’, peak late diastolic mitral annular velocity by tissue Doppler; e’, peak early diastolic mitral annular velocity by tissue Doppler; EF, ejection fraction; dP/dtmax, the maximal slope of LV systolic pressure increment; dP/dtmin, the maximal slope of LV diastolic pressure decrement; Tau (τ), the time constant for isovolumic relaxation; DT, E wave deceleration time; sGC, soluble guanylyl cyclase. Data are shown as mean ± SEM; *P < 0.05, ^∗^^∗^*P < 0.001*,*^∗∗^P < 0.0001 control untreated versus DSS untreated and ^†^P < 0.05, ^†⁣†^P < 0.001, ^†⁣†⁣†^P < 0.0001 before versus after sGC activator treatment.

### Improved Cardiomyocyte Function Upon Soluble Guanylyl Cyclase Activator Treatment in Dahl Salt-Sensitive Rats

**FIGURE 1:**
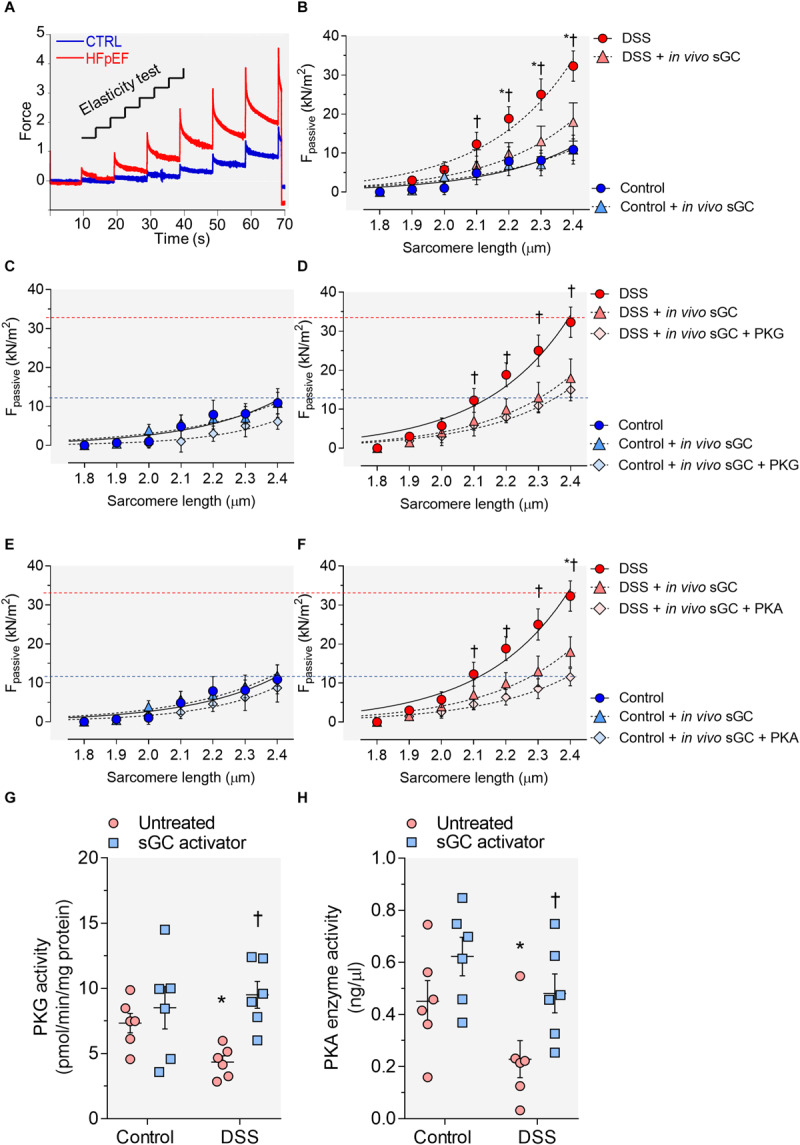
Cardiomyocyte passive stiffness and protein kinase (PK)G and PKA activity in the rat model. **(A)** Original recording of the force response to stepwise cell stretching of isolated skinned cardiomyocytes. **(B)** Control and Dahl salt-sensitive (DSS) passive force (F_passive_) at sarcomere length (SL) 1.8–2.4 μm in the presence or absence of *in vivo* soluble guanylyl cyclase (sGC) activator. **(C,D)** Control and DSS F_passive_ at SL 1.8–2.4 μm in the presence or absence of *in vivo* sGC activator and subsequently added PKG. **(E,F)** Control and DSS F_passive_ at SL 1.8–2.4 μm in the presence or absence of *in vivo* sGC activator and subsequently added PKG. Curves are second-order polynomial fits to the means (± SEM; *n* = 4–5 cardiomyocytes/heart). For **(B)**, *P < 0.05 control baseline versus DSS baseline, ^†^*P* < 0.05 DSS baseline versus DSS after sGC activator treatment. **(D)** *P < 0.05 DSS baseline versus DSS after sGC activator treatment, ^†^*P* < 0.05 DSS after sGC activator treatment versus DSS after sGC activator treatment followed by PKG treatment. **(F)** *P < 0.05 DSS baseline versus DSS after sGC activator treatment followed by PKA in Student’s *t-*test. **(G)** PKG activity. **(H)** PKA activity. Data are shown as mean ± SEM; *n* = 7–8 left ventricular (LV) samples/group. *P < 0.05 control untreated versus DSS untreated and ^†^*P* < 0.05 before versus after sGC activator treatment.

To assess the functional effects of sGC activator, we measured cardiomyocyte stiffness (F_passive_). Figure 1A shows a representative elasticity test protocol and force recording for both DSS rats and controls. DSS rats showed a steeper F_passive_ compared to controls (Figure 1B). Acute *in vivo* treatment with sGC activator significantly reduced cardiomyocyte F_passive_ at SLs 2.1–2.4 μm (Figures 1D,F), but did not affect controls. We then treated cardiomyocytes with either PKG or PKA to determine whether these two kinases have an effect additional to sGC activator. Supplementary PKG (Figures 1C,D) and PKA (Figures 1E,F) had no significant effect on F_passive_ in sGC activator-treated DSS rats as was also the case for the sGC activator-treated control group. PKG and PKA activity was significantly reduced in DSS rats and was restored to control levels after treatment with sGC activator (Figures 1G,H).

### Improved Titin Phosphorylation After Soluble Guanylyl Cyclase Activator Treatment

**FIGURE 2:**
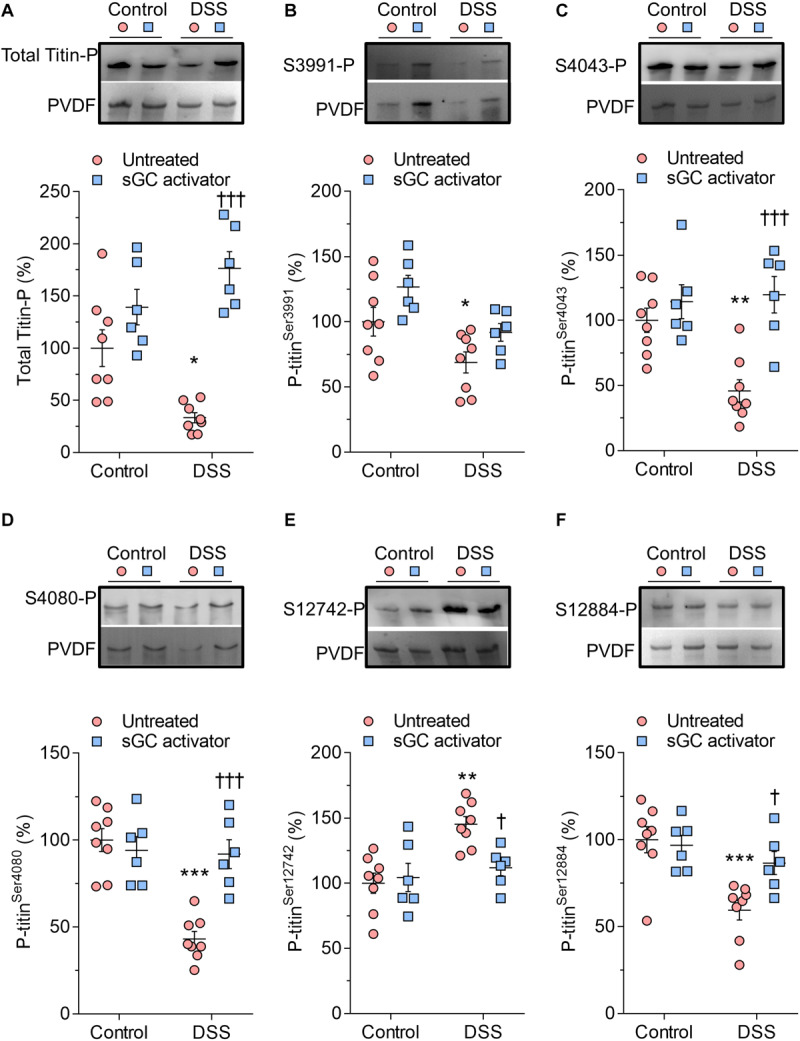
Titin phosphorylation in Dahl salt-sensitive (DSS) and control rats. **(A)** Total titin phosphorylation. **(B)** Phospho (P) site-specific within titin-N2Bus at Ser3991. **(C)** P site-specific within titin-N2Bus at Ser4043. **(D)** P site-specific within titin-N2Bus at Ser4080. **(E)** P site-specific within titin PEVK segment at Ser-12742. **(F)** P site-specific within titin PEVK segment at Ser-12884. Data are shown as mean ± SEM; *n* = 7–8 left ventricular (LV) samples/group. *P < 0.05, **P < 0.001, ***P < 0.0001 control untreated versus DSS untreated and ^†^*P* < 0.05, ^†⁣†⁣†^*P* < 0.0001 before versus after soluble guanylyl cyclase (sGC) activator treatment.

Cardiomyocyte F_passive_ is partially determined by titin phosphorylation, an important downstream target of the cGMP-PKG and cAMP-PKA pathways. We therefore studied whether improved cardiomyocyte stiffness is due to changes in titin phosphorylation. We assessed total titin using a Ser/Thr antibody and measured conserved phosphoserines within the I-band region, including three serines within the N2Bus (Ser3991, Ser4043, and Ser4080 of full-length mouse titin) and conserved serines in the PEVK segment (Ser12742 and Ser12884). Total titin phosphorylation was significantly reduced in DSS animals and restored upon sGC activator treatment (Figure 2A). Phosphoserines located within the I-band region (Ser3991, Ser4043, Ser4080), together with Ser12884 in the PEVK segment, were hypo-phosphorylated (Figures 2B–D,F), while Ser12742 of the PEVK domain was hyperphosphorylated, in DSS rats. sGC activator significantly increased the phosphorylation of Ser4043, Ser4080, and Ser12884 but did not affect the phosphorylation of Ser3991 (Figures 2B–D,F) and significantly reduced Ser12742 phosphorylation (Figure 2E).

### Kinases Activity Upon Treatment With Soluble Guanylyl Cyclase Activator in Dahl Salt-Sensitive Rats

**FIGURE 3:**
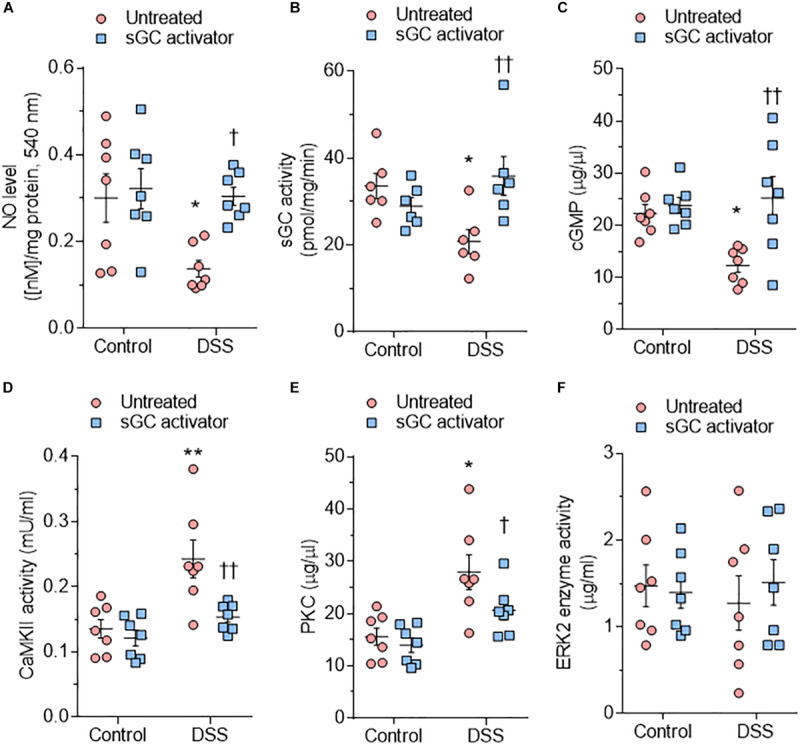
Soluble guanylyl cyclase (sGC)–cyclic guanosine monophosphate (cGMP) pathway components and activities of major kinases in the rat model. **(A)** Nitric oxide (NO) bioavailability. **(B)** sGC activity. **(C)** Myocardial cGMP-dependent protein kinase (PK) concentration. **(D)** Calcium/calmodulin-dependent kinase II (CaMKII) activity. **(E)** PKC activity. **(F)** Extracellular signal-regulated kinase 2 (ERK2) activity. Data are shown as mean ± SEM; *n* = 6–7 left ventricular (LV) samples/group. *P < 0.05, **P < 0.001, ***P < 0.0001 control untreated versus Dahl salt-sensitive (DSS) untreated and ^†^*P* < 0.05, ^†⁣†^*P* < 0.001 before versus after sGC activator treatment.

Bioavailability of NO is reduced by oxidative stress and impacts both the activity and concentration of the downstream targets sGC and cGMP (Franssen et al., 2016). In DSS rats, NO bioavailability, sGC activity, and cGMP concentration were reduced at baseline and restored after sGC treatment (Figures 3A–C). To understand the beneficial effect of sGC on signaling pathways and thereby on cardiomyocyte function, we assessed protein kinases, including CaMKII, PKC, and ERK2, that play a role in maladaptive remodeling of HFpEF myocardium. The activities of CaMKII and PKC were significantly high in DSS rats and reduced upon treatment with sGC activator, whereas ERK2 activity did not differ between DSS rats and controls and remained unaltered after the treatment (Figures 3D–F). Kinase activities did not show any changes between controls and controls treated with sGC activator.

### Soluble Guanylyl Cyclase Activator Promotes Translocation of Soluble Guanylyl Cyclase Toward the Intercalated Disc Region

**FIGURE 4:**
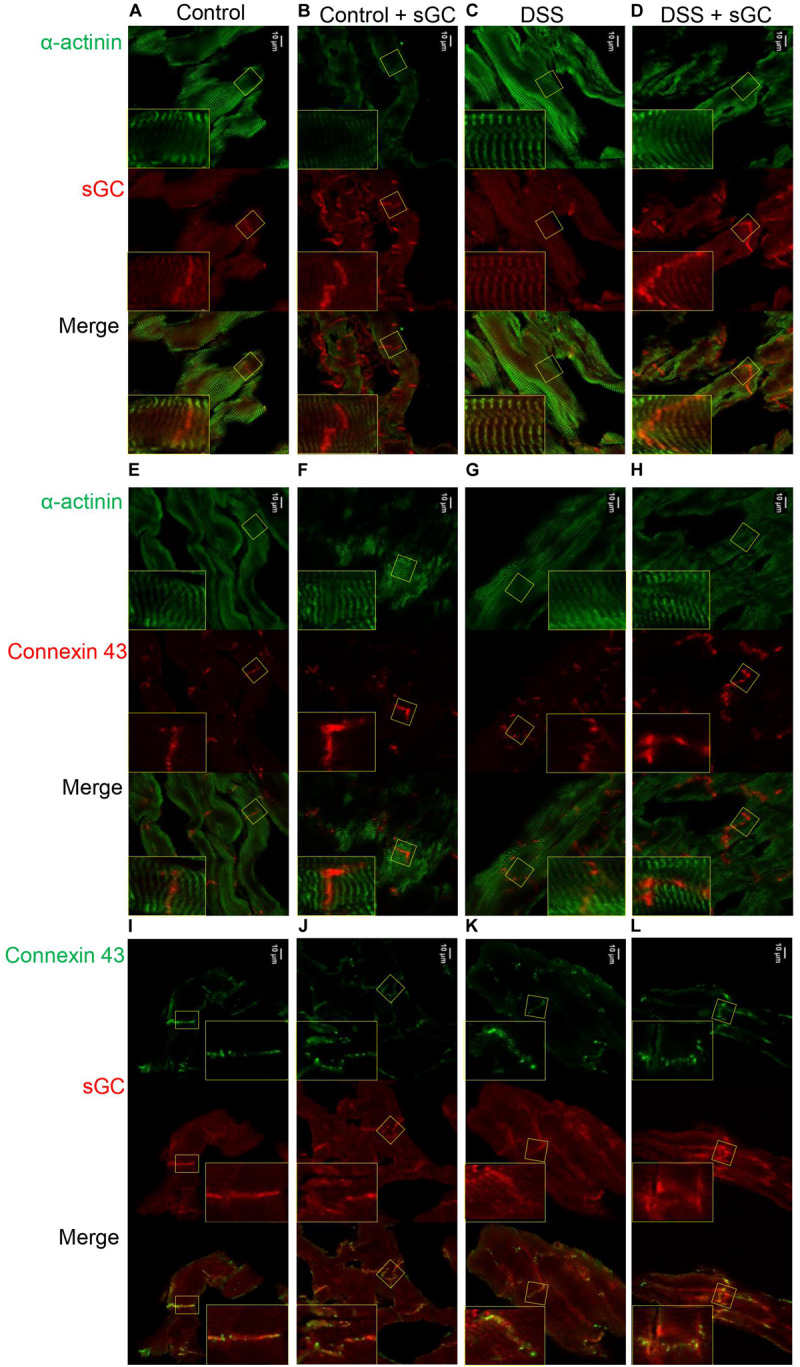
cLSM images of control and DSS rat cardiomyocytes before and after sGC activator treatment demonstrating sGC translocation to the intercalated disc **(A–D)**. Representative immunofluorescence images of cardiomyocytes stained for cardiac sGC (red) and counterstained for α-actinin (green). **(E–H)** Immunofluorescence images of cardiomyocytes stained for connexin 43 (red) and counterstained for α-actinin (green). **(I–L)** Representative immunofluorecence images of cardiomyocytes stained for connexin 43 (green) and sGC (red).

**FIGURE 5:**
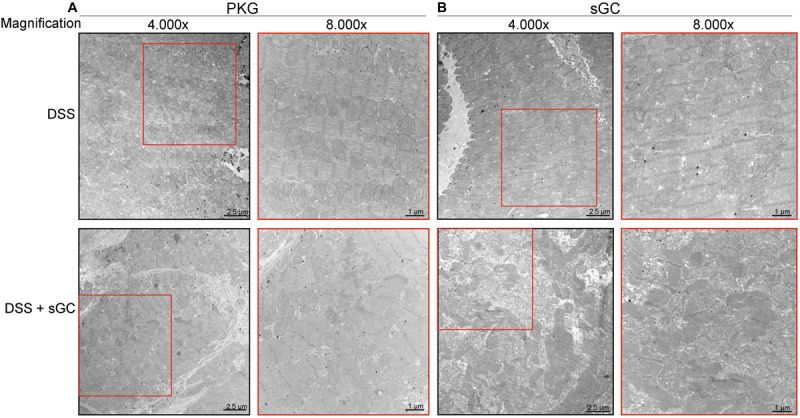
Immunoelectron micrographs of Dahl salt-sensitive (DSS) rat cardiomyocytes before and after soluble guanylyl cyclase (sGC) activator treatment stained for protein kinase (PK)G or sGC. **(A)** Representative immunoelectron micrographs of cardiomyocytes from DSS + DSS-treated rat hearts stained for PKG. **(B)** Immunoelectron micrographs of cardiomyocytes from DSS + DSS-treated rat hearts stained for sGC.

Using confocal microscopy, we found that sGC is distributed throughout the cytosol and intercalated disc in control rats (Figure 4A), whereas sGC appeared to be more concentrated in the myofilaments and almost absent around the intercalated disc in DSS rats (Figure 4C). Treatment with sGC activator promoted the translocation of sGC from the cytosol to the intercalated disc, as seen in Figures 4B,D. We confirmed translocation to the intercalated disc by staining for connexin 43, an integral gap junction protein that is part of the intercalated disc and t-tubuli. Furthermore, a disrupted pattern and reduced level of connexin 43 was noted in DSS rats, and the pattern partially improved after sGC activator treatment in both control treated and DSS treated rats (Figures 4E–H). We also performed a dual staining of connexion 43 and sGC to show the translocation of sGC toward the intercalated disc region (Figures 4I–L). As shown, there is a localization of connexion 43 and sGC in all groups. However, in the treated groups control and DSS clearly show enhanced presence of sGC in the intercalated disc region, confirming the previous suggested translocation of the sGC toward the intercalated disc region. Electron microscopy showed lower expression of PKGIα and sGC proteins in DSS rats, and specifically in cardiomyocytes, while expression was enhanced in cardiomyocytes after sGC treatment (Figures 5A,B).

### Pro-Inflammatory Cytokines and Oxidative Stress Are Reduced in Dahl Salt-Sensitive Rats

**FIGURE 6:**
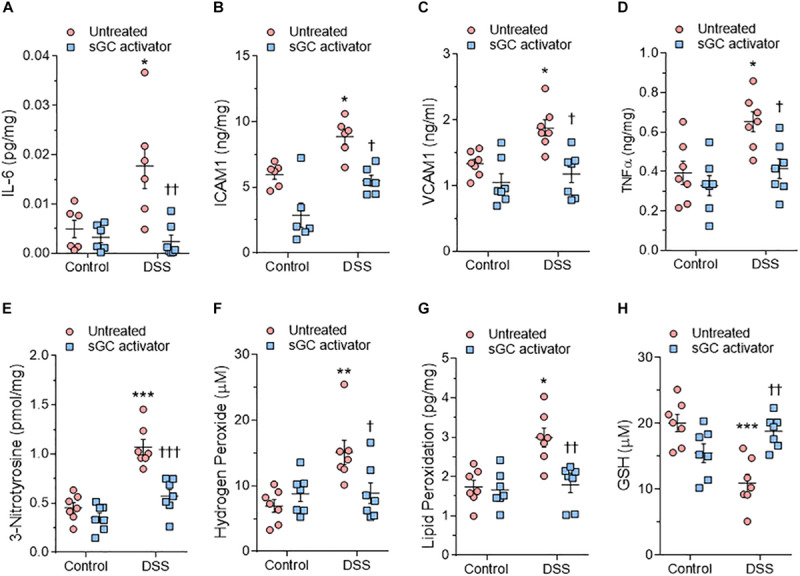
Myocardial inflammation and oxidative stress in heart tissue from the rat model. **(A)** Interleukin 6 (IL-6). **(B)** Intercellular adhesion molecule 1 (ICAM1). **(C)** Vascular cell adhesion molecule 1 (VCAM1). **(D)** Tumor necrosis factor alpha (TNFα). **(E)** 3-nitrotyrosine. **(F)** Hydrogen peroxide (H_2_O_2_). **(G)** Lipid peroxide (LPO). **(H)** Reduced glutathione (GSH). Data are shown as mean ± SEM; *n* = 7–8 left ventricular (LV) samples/group. *P < 0.05, **P < 0.001, ***P < 0.0001 control untreated versus Dahl salt-sensitive (DSS) untreated and ^†^*P* < 0.05, ^†⁣†^*P* < 0.001, ^†⁣†⁣†^*P* < 0.0001 before versus after sGC activator treatment.

We used ELISA to assess LV myocardial pro-inflammatory cytokines including interleukin-6 (IL6), intercellular cell adhesion molecule-1 (ICAM1), vascular cell adhesion molecule-1 (VCAM1), and tumor necrosis factor alpha (TNFα), which were all found to be elevated in DSS rats compared to the control group (Figures 6A–D). This upregulation was reduced to control group levels upon treatment with sGC activator, but no effect was found in the control group after treatment with sGC (Figures 6A–D). To determine oxidative stress levels, we measured 3-nitrotyrosine, lipid peroxide (LPO), H_2_O_2_, and reduced glutathione (GSH), all of which were found to be significantly upregulated in DSS rats compared to the control group. Treatment with sGC activator reduced oxidative stress parameters to control group levels (Figures 6E–H).

### Improved Passive Force Upon Treatment With Soluble Guanylyl Cyclase Activator in Human Heart Failure With Preserved Ejection Fraction

**FIGURE 7:**
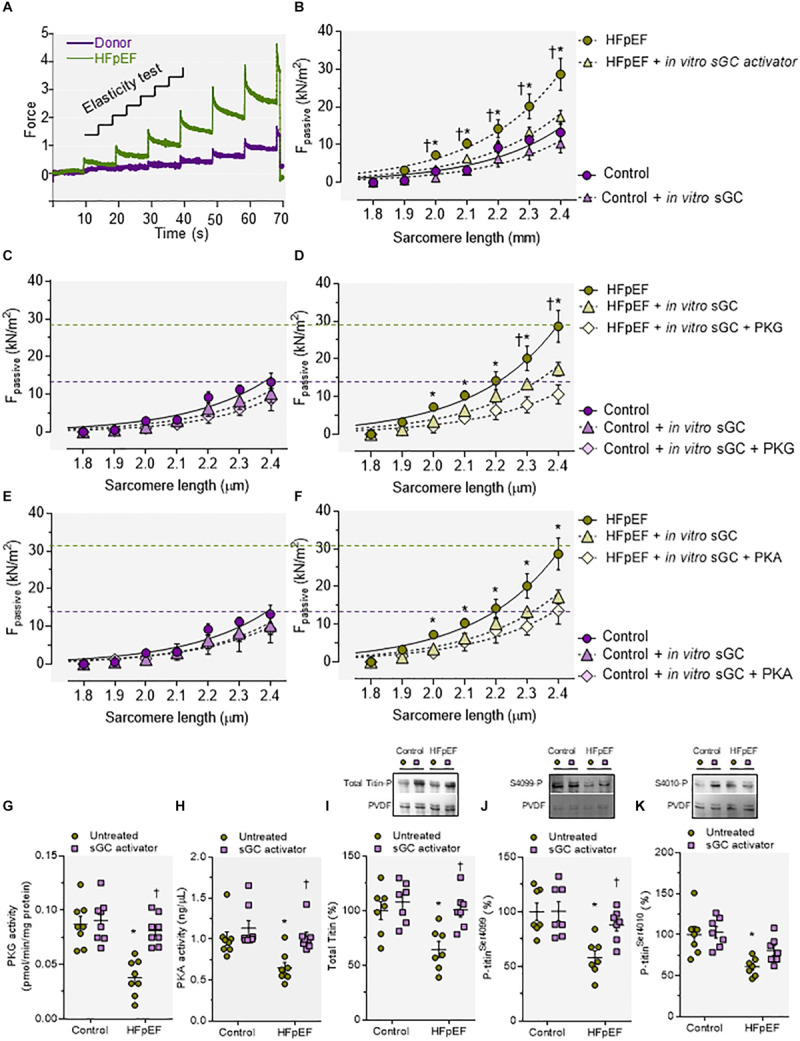
Cardiomyocyte passive stiffness; protein kinase (PK)G and PKA activities and titin phosphorylation of myocardial biopsies of human heart failure with preserved ejection fraction (HFpEF) patients before and after *in vitro* incubation with soluble guanylyl cyclase (sGC) activator. **(A)** Original recording of the force response to stepwise cell stretching of isolated skinned cardiomyocytes. **(B)** Control and Dahl salt-sensitive (DSS) passive force (F_passive_) at sarcomere length (SL) 1.8–2.4 μm in the presence or absence of sGC activator. **(C,D)** Control and DSS F_passive_ at SL 1.8–2.4 μm in the presence or absence of sGC activator and subsequently added PKG. **(E,F)** Control and DSS F_passive_ at SL 1.8–2.4 μm in the presence or absence of sGC activator and subsequently added PKA. Fit curves are two-order polynomials to the means. Data are mean ± SEM; *n* = 4–5/heart. For **(B)**, *P < 0.05 control baseline versus HFpEF baseline, ^†^*P* < 0.05 HFpEF baseline versus after sGC activator treatment. **(D)** *P < 0.05 HFpEF baseline versus HFpEF after sGC activator treatment, ^†^*P* < 0.05 HFpEF after sGC activator treatment versus HFpEF after sGC activator treatment followed by PKG treatment. **(F)** *P < 0.05 HFpEF baseline versus HFpEF after sGC activator treatment in Student’s *t*-test. **(G)** PKG activity. **(H)** PKA activity. **(I)** Total titin phosphorylation. **(J)** Site-specific phosphorylation of titin-N2Bus at Ser4099. **(K)** Site-specific phosphorylation of titin-N2Bus at Ser4010. Data are shown as mean ± SEM; *n* = 7–8 left ventricular (LV) samples/group. *P < 0.05 control untreated versus HFpEF untreated and ^†^*P* < 0.05 before versus after sGC activator treatment.

To study the functional effect of sGC activator in human myocardial biopsies (Table 1) from HFpEF patients we measured cardiomyocyte F_passive_ before and after acute *in vitro* treatment. Figure 7A shows a representative elasticity test protocol and force recording for HFpEF myocardium biopsies and controls. Human HFpEF myocardium biopsies showed increased cardiomyocyte F_passive_ compared to controls, which confirmed previous findings (Figure 7B) (Borbely et al., 2005; Franssen et al., 2016). This increase could be reduced upon *in vitro* treatment with sGC activator (Figure 7D), while no changes were observed in control non-failing donor hearts after treatment (Figure 7C). Additional treatment with PKGIα further significantly reduced F_passive_ at SL 2.3 and 2.4 μm, indicating that the sGC activator did not fully correct the increased cardiomyocyte Fpassive observed in HFpEF myocardium biopsies, perhaps due to the fact that PKGIα did not fully phosphorylate titin-mediated PKG phosphorylation, while PKA had no further significant effect after sGC treatment (Figures 7E,F). To assess whether these functional changes could be due to the level of PKG and PKA activity, we measured the activity of these kinases and found both to be significantly depressed in HFpEF myocardium biopsies. sGC significantly restored activity (Figures 7G,H). Improved cardiomyocyte function was related to improved total titin phosphorylation, which was originally reduced in these biopsies (Figure 7I), in addition to increased phosphorylation of the specific serine phospho-sites Ser4010 (PKA-dependent) and Ser4099 (PKG-dependent) of full-length human titin in DSS rats after sGC treatment. Both phosphosites were significantly downregulated at the baseline before the treatment. (Figures 7J,K)

### Soluble Guanylyl Cyclase Activator Improves Kinases Involved in Hypertrophic Pathways and Reduces Pro-Inflammatory Cytokines and Oxidative Stress in Human Heart Failure With Preserved Ejection Fraction

**FIGURE 8:**
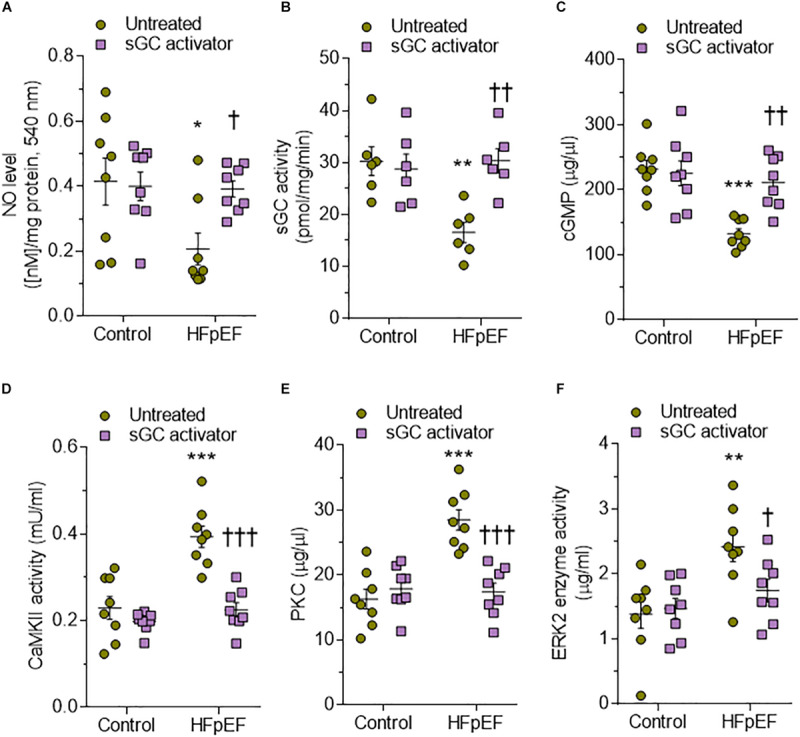
Soluble guanylyl cyclase (sGC)–cyclic guanosine monophosphate (cGMP) pathway components and activities of major kinases in human heart failure with preserved ejection fraction (HFpEF) hearts. **(A)** Nitric oxide (NO) bioavailability. **(B)** sGC activity. **(C)** Myocardial cGMP-dependent protein kinase levels. **(D)** Calcium/calmodulin-dependent kinase II (CaMKII) activity. **(E)** Protein kinase C (PKC) activity. **(F)** Extracellular signal-regulated kinase 2 (ERK2) activity. Data are shown as mean ± SEM; *n* = 7–8 left ventricular (LV) samples/group. *P < 0.05 control untreated versus HFpEF untreated and ^†^*P* < 0.05 before versus after sGC activator treatment.

**FIGURE 9:**
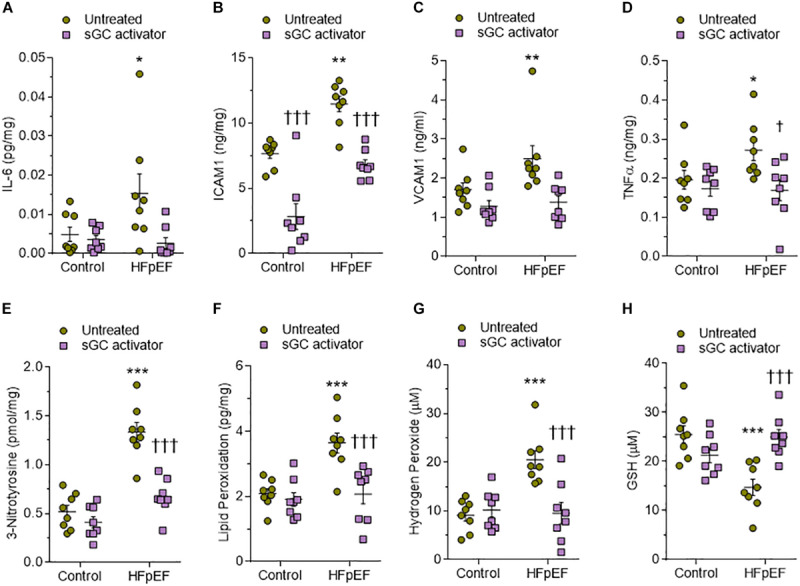
Expression of markers of myocardial inflammation and oxidative stress in non-failing and heart failure with preserved ejection fraction (HFpEF) patient hearts. **(A)** Interleukin 6 (IL6). **(B)** Intercellular adhesion molecule 1 (ICAM1). **(C)** Vascular cell adhesion molecule 1 (VCAM1). **(D)** Tumor necrosis factor alpha (TNFα). **(E)** 3-nitrotyrosine. **(F)** Hydrogen peroxide (H_2_O_2_). **(G)** Lipid peroxide (LPO). **(H)** Reduced glutathione (GSH). Data are shown as mean ± SEM; *n* = 7–8 left ventricular (LV) samples/group. *P < 0.05 control untreated versus HFpEF untreated and ^†^*P* < 0.05 before versus after soluble guanylyl cyclase (sGC) activator treatment.

Human HFpEF myocardium biopsies showed a trend similar to that observed in DSS rats, with a significant reduction of the sGC–cGMP–PKG pathway that could be attenuated upon treatment with sGC activator (Figures 8A–C). CaMKII, PKC, and ERK2 were significantly upregulated in HFpEF myocardium biopsies compared to non-failing donor hearts and were lowered by treatment with sGC activator (Figures 8D–F). We then assessed whether pro-inflammatory cytokines (IL-6, ICAM1, VCAM1, TNFα) and oxidative stress (3-nitrotyrosine, LPO, H_2_O_2_, GSH) were higher in HFpEF human myocardium biopsies and studied the effect of *in vitro* sGC activator treatment on these parameters. As expected, *in vitro* treatment with sGC activator significantly reduced inflammatory cytokines and oxidative stress to the levels observed in control non-failing donor hearts (Figures 9A–H). The non-failing donor hearts showed no changes in inflammatory cytokines or oxidative stress after sGC treatment (Figures 9A–H).

## Discussion

Currently, limited understanding of HF with LV diastolic dysfunction is one of the major reasons for the inability to develop effective treatment options for patients with this life-threatening condition. A more modern updated view considers a complex interaction of structural, functional, molecular, and organismal alterations that account for the progressive nature of this HF type. Metabolic disorders such as obesity, metabolic syndrome, and diabetes mellitus are major cardiovascular risk factors, are accompanied by diastolic dysfunction and an increased risk of mortality, and are often associated with oxidative stress (Paulus and Tschope, 2013; Franssen et al., 2016). Recent evidence by us and others has suggested that oxidative stress may be the mechanistic link between obesity, diabetes mellitus, and related complications (Borlaug and Paulus, 2011; Paulus and Tschope, 2013; Franssen et al., 2016). Earlier failures to identify the underlying causes of the syndrome may explain the disappointing clinical trial results and indicate that progress will not be possible until we disentangle symptoms from causes. Our current findings show that the sGC activator BAY 58-2667 improves cardiomyocyte function *via* improved titin phosphorylation, an effect likely due to improved signaling pathways including the pathway NO–sGC–cGMP–PKG and the hypertrophic pathways mediated by CaMKII, PKC, and ERK2, in addition to the PKA pathway. Moreover, the improvement mediated by sGC activation also seems to be related to the reorganization and increased expression of connexin 43 at the gap junctions in both control and DSS rats. Interestingly, translocation of sGC toward the intercalated disk was observed in controls and DSS rats after the treatment. Finally, improved cardiomyocyte function after sGC treatment appears to be associated with reduced pro-inflammatory cytokines and oxidative stress parameters.

An earlier study showed that in the myocardium of HFpEF patients and ZSF1-HFpEF rats, E-selectin, ICAM, and VCAM expression levels were upregulated, in addition to raised expression of nicotinamide adenine dinucleotide phosphate (NADPH) oxidase 2 in macrophages and endothelial cells but not in cardiomyocytes (Franssen et al., 2016). In addition, the uncoupling of endothelial NO synthase was associated with reduced myocardial nitrite/nitrate concentration, cGMP content, and PKG activity. Taken together, these findings indicate that increased inflammation and oxidative stress contribute to the reduced NO-dependent signaling from endothelial cells to cardiomyocytes and to the high cardiomyocyte stiffness and hypertrophy observed in HFpEF (Franssen et al., 2016) and in the current study. TNFα and IL-1β further promote cardiomyocyte hypertrophy (Yokoyama et al., 1997), and the cytokine IL-6 can increase cardiomyocyte stiffness *via* a reduction in titin phosphorylation (Savvatis et al., 2014).

Reduced NO signaling is resulting from increased generation of reactive oxygen species (ROS), endothelial damage, and NO synthase impairment but also reduced NO binding to sGC due to oxidative stress. This interruption of NO signaling can lead to endothelial dysfunction, cardiomyocyte dysfunction, fibrosis, and inflammation (Franssen et al., 2016). sGC is a key signal transduction enzyme in the cardiovascular system and is not only impaired in HFpEF but also in a variety of diseases such as hypertension, pulmonary hypertension, chronic kidney disease, and erectile dysfunction, all of which are associated with impaired NO–sGC–cGMP signaling (Kemp-Harper and Feil, 2008; Schulz et al., 2008; Stasch et al., 2011; Klinger and Kadowitz, 2017). sGC activators have a beneficial effect on cardiomyocyte function in DSS rats and HFpEF myocardium biopsies, which could be beneficial for diastolic dysfunction, as observed in a study by Esposito et al. (2017), and could therefore represent a potential HFpEF treatment. Cardiomyocyte improvement in both DSS rats and HFpEF human myocardium appears to be associated with an improved NO–sGC–cGMP–PKG pathway, in addition to the PKA pathway and hypertrophic pathways such as CaMKII, ERK2, and PKC. Therefore, an improved NO–sGC–cGMP–PKG pathway could be the result of improved sGC bioavailability due to activation of sGC or the result of reduced oxidative stress and inflammation, which may have affected endothelial function and cardiomyocyte function *via* the increased NO bioavailability observed in DSS rats and HFpEF myocardium biopsies.

Cardiomyocyte stiffness is mainly determined by titin isoform switching, titin phosphorylation, and titin oxidation (Alegre-Cebollada et al., 2014; Linke and Hamdani, 2014). Previously, it was shown that titin is phosphorylated by different kinases, with distinct effects on cardiomyocyte F_passive_ (Yamasaki et al., 2002; Fukuda et al., 2005; Kruger and Linke, 2006; Borbely et al., 2009; Hidalgo et al., 2009; Kruger et al., 2009; Raskin et al., 2012). The cardiac titin N2Bus spring element is phosphorylated by PKA (Yamasaki et al., 2002; Fukuda et al., 2005; Kruger and Linke, 2006), PKG (Kruger et al., 2009), ERK2 (Raskin et al., 2012), and CaMKIIδ (Hamdani et al., 2013b). All of these kinases reduce F_passive_ (Yamasaki et al., 2002; van Heerebeek et al., 2006; Borbely et al., 2009; Kruger et al., 2009; Raskin et al., 2012). By contrast, a PEVK region constitutively expressed in full-length titin is phosphorylated by PKCα, and this modification increases cardiomyocyte F_passive_ (Hidalgo et al., 2009). Thus, acute changes in titin-based stiffness *via* titin phosphorylation can occur in opposite directions, depending on which signaling pathway is activated (Linke and Hamdani, 2014). The current study shows that acute treatment with sGC activator increases total titin phosphorylation and site-specific phosphorylation of titin, specifically at the elastic regions N2Bus and PEVK, in turn decreasing titin-based cardiomyocyte passive stiffness in both DSS rats and HFpEF myocardium biopsies. We also showed improved site-specific phosphorylation at the elastic N2Bus titin site, indicating that improved titin phosphorylation results from improvements in signaling pathways involving PKA, PKG, CaMKII, and/or ERK2. We previously reported evidence that activation of cGMP–PKG in a large animal model of HFpEF treated acutely with sildenafil followed by BNP led to increased titin phosphorylation, lower cardiomyocyte F_passive_, and improved diastolic function (Bishu et al., 2011). However, in other large animal models, acute stimulation of sGC did not impact LV capacitance, for example, in normal and hypertrophied porcine hearts *in vivo* (Alogna et al., 2018). Nevertheless, titin phosphorylation was increased, suggesting that in this model, increased titin phosphorylation is perhaps not indicative of increased *in vivo* LV capacitance (Alogna et al., 2018). Similarly, an improved cGMP–PKG signaling pathway has been demonstrated in a small animal model with diastolic dysfunction treated chronically with DPP4 inhibitor (an enzyme involved in cGMP degradation). This model showed improved diastolic stiffness and cardiomyocyte function through increased titin phosphorylation and reduced fibrosis (Hamdani et al., 2014).

In the present study, we found reduced PKC activity and improved titin–PEVK site-specific phosphorylation after treatment with the sGC activator, both of which may have contributed to the reduced cardiomyocyte stiffness. Additional posttranslational modifications that alter the stiffness of titin include arginylation (Leite Fde et al., 2016) and various oxidative modifications, such as disulfide bonding (Grutzner et al., 2009; Giganti et al., 2018), S-glutathionylation (Alegre-Cebollada et al., 2014), and sulphenylation (Beedle et al., 2016). Therefore, since the sGC activator reduces oxidative stress, improved cardiomyocyte stiffness could be related, in part, to reduced titin oxidation. The reversible oxidative modifications previously found in the various I-band titin regions have been shown to modify titin-based stiffness also in isolated human cardiomyocytes (Linke and Hamdani, 2014; Beckendorf and Linke, 2015; Breitkreuz and Hamdani, 2015). Disulfide bond formation in the cardiac-specific N2-Bus region appeared to decrease N2B extensibility, stiffen the titin molecule, and increase cardiomyocyte F_passive_ (Grutzner et al., 2009). In contrast, cysteine S-glutathionylation in the Ig domains of the elastic I-band region affects cardiomyocyte F_passive_ in the opposite direction (Alegre-Cebollada et al., 2014). Explanation of this effect considers that cryptic cysteines in titin Ig domains become available for redox modification when they unfold. Upon stretching of cardiac sarcomeres, Ig domain unfolding increases and cryptic cysteines becoming exposed to oxidized glutathione will be S-glutathionylated, which greatly weakens the mechanical stability of the Ig domains and their ability to refold, leading to a reduced cardiomyocyte F_passive_ (Alegre-Cebollada et al., 2014). This effect can be reversed by adding reduced glutathione (Alegre-Cebollada et al., 2014). Conversely, S-sulphenylation of cryptic cysteines in Ig domains can cause titin stiffening by promoting S-S bonding in titin Ig domains (Beedle et al., 2016). Taken together, the contribution of titin oxidation to cardiomyocyte F_passive_ modulation cannot be discounted in the current study. In addition to titin, we also checked the phosphorylation status of calcium handling proteins including ryonidine receptor (RYR) and phospholamban (PLN) at serines Ser2808 and Ser4080, respectively. RyR phosphorylation was unaltered in DSS rats and was comparable to the controls. sGC activator did not further affect both groups. Phosphorylation of PLN was not significantly changed in DSS rats compared to controls, and sGC activator significantly increased the baseline phosphorylation level in both control and DSS groups. This suggested perhaps also the contribution of PLN in improved cardiac function.

In addition, NO may also promote protein modification through nitration of tyrosines to form the stable 3-nitrotyrosine. Accumulation of 3-nitrotyrosine in proteins is indicative of ROS and reactive nitrogen species (RNS) stress (Ischiropoulos, 2003). When redox-active thiols are combined with RNS in our system, it forms S-nitrosothiols, which then alters protein function. The protein thiol redox state of cells is maintained by the mitochondria, which are a major source of cellular oxidants, and the basal production of oxidants. This in turn affects both global S-nitrosation and disulfide formation of proteins (Ischiropoulos, 2003). Therefore, we cannot exclude that the sGC activator in our model might have an effect on the mitochondrial function and might also contribute to pathway improved function seen in the DSS treated rats.

The sGC activator induced a reversible shift toward pro-inflammatory cytokines and reduced oxidative stress, influencing cardiomyocyte function *via* improved signaling pathways and thereby titin phosphorylation. Cardiac fibrosis is also involved in the modulation of diastolic function and is an important contributor to diastolic impairment in HFpEF as it increases LV and peripheral vascular stiffness (Borbely et al., 2005; van Heerebeek et al., 2008; Zile et al., 2015), an impairment due to the migration of leukocytes and macrophages in humans (Kai et al., 2005) and experimental HF models (Luft et al., 1999; Fischer et al., 2008; Haase et al., 2014). BAY 58-2667 has been shown to reduce monocyte/macrophage infiltration into cardiac tissue and to reverse inflammatory gene expression patterns in failing rat hearts (Wilck et al., 2018), in addition to its role in reducing interstitial fibrosis and blood pressure (Masuyama et al., 2006); these combined actions likely result in improved diastolic function.

Strikingly, improved cardiomyocyte function could be attributed, in part, to sGC translocation, as immunohistochemistry found sGC preferentially at the intercalated disk after treatment of DSS rats with sGC activator, whereas the sGC signal was almost absent from the intercalated disk in DSS rats before treatment. In addition, sGC appeared to have translocated somewhat more to the sarcomere after treatment of DSS cardiomyocytes with the activator. Interestingly, connexin 43 appeared to be reduced and disrupted at the intercalated disk in DSS rats, an effect partially reversed after treatment. Taken together, these findings suggest that a functional NO–sGC–cGMP pathway preserves the intercalated disk, the site of mechanical and electrical conduction between cardiomyocytes and perhaps also fibroblasts (Menges et al., 2019) and endothelial cells (Yuan et al., 2015; Johnson and Camelliti, 2018). Connexin 43 expressed in endothelial cells modulates monocyte–endothelial adhesion by regulating cell adhesion proteins, an interaction that is decreased upon reduced connexin 43 (Yuan et al., 2015). Furthermore, in connexin 43-knockout mice, ischemia leads to an increased frequency and duration of ventricular tachycardia as well as spontaneous ventricular arrhythmia, which results in higher rates of sudden cardiac death (Johnson and Camelliti, 2018). Formation of connexin 43-containing gap junctions at the intercalated disk involves trafficking from the endoplasmic reticulum to the Golgi to stabilize the intercalated disk (Thevenin et al., 2013). This process may be controlled by kinase activation that leads to connexin 43 phosphorylation, in addition to interaction with several additional binding partners. As it has been proposed that PKA may be involved in connexin 43–gap junction assembly (Solan and Lampe, 2007, 2014), we speculate that PKG may also be involved. cAMP and cGMP are ubiquitous second messengers with a similar range of functions in vascular homeostasis and disease. However, the exact roles of both molecules in the regulation of connexins and gap junction intercellular communication are still unclear, although it is known that activation of the NO–sGC–cGMP induces connexin 43 expression and increases intercellular communication *via* gap junctions (Yao et al., 2005). Our observation that sGC is located at the intercalated disk and that this localization is disrupted in DSS rats, together with the novel finding that connexin 43 is also disrupted and reduced at the intercalated disk in DSS rats suggests that further investigation of the potential function of the NO–sGC–cGMP pathway in relation to connexin 43 function is warranted in HFpEF patients.

Numerous studies have demonstrated the cardioprotective effects of NO–sGC–cGMP–PKG signaling. sGC stimulation can attenuate LV remodeling after myocardial infarction in mice (Frankenreiter et al., 2018; Berg et al., 2019), decrease extracellular matrix protein production in human cardiac fibroblasts following TGF-β stimulation, and attenuate vascular dysfunction in diabetic rats (Grontved et al., 2014). The eNOS transcriptional enhancer (AVE9488) improves cardiac remodeling after myocardial infarction (Fraccarollo et al., 2008) and platelet NO availability and hyperactivity in HF. Our studies indicate that the sGC activator can act in a similar fashion under oxidative stress, and inflammation was reduced in our animal model and in HFpEF myocardium biopsies after treatment. We have shown that sGC activator can positively impact the pathology of DSS rats with diastolic dysfunction and in HFpEF myocardium human biopsies *via* improved cardiomyocyte function.

## Conclusion

Our data show that an sGC activator improves cardiomyocyte function, reduces inflammation and oxidative stress, and improves NO–sGC–cGMP–PKG signaling and hypertrophic signaling pathways including CaMKII, ERK2, and PKCα. These findings suggest that upstream reduction of inflammation and oxidative stress, together with the enhancement of signaling pathways by sGC activators, may provide new opportunities for improving diastolic function in HFpEF patients.

## Data Availability Statement

All datasets generated for this study are included in the article/supplementary material.

## Ethics Statement

The studies involving human participants were reviewed and approved by Berlin, Germany Ethics Committee approval (EA2/140/16). The patients/participants provided their written informed consent to participate in this study. The animal study was reviewed and approved by Ruhr University Bochum (Germany; ethical statement number: 84-02.04.2015.A449).

## Author Contributions

DK has performed all experiments, analyzed all data, and wrote the manuscript. ÁK performed mechanics experiments and *in vivo* study with sGC activator. MH performed biochemistry experiments and wrote the manuscript. ML performed electron microscopy. MS performed biochemistry experiments. AA performed electron microscopy. PS provided the drug and revised the manuscript. ZP helped with the mechanic experiments. PR revised the manuscript. PH provided tissues and revised the manuscript. IF-P supervised *in vivo* study. WL helped with interpretation of the data and rewrote the manuscript. KJ supervised the biochemistry experiments. SV and CT provided tissues, analyzed all clinical data, and revised the manuscript. AM revised the manuscript. NH designed the study, supervised all experiments, performed mechanics, analyzed all data, and wrote the manuscript.

## Conflict of Interest

The authors declare that this study received funding from Bayer AG. The funder was not involved in the study design, collection, analysis, interpretation of data, the writing of this article or the decision to submit it for publication. Howere, the funder had the following involvement with the study “Approval of the manuscript.”
